# Life and Writings of Botalli

**Published:** 1861-09

**Authors:** 


ART. II.—*Leonardi Botalli Astensis, Philosophiæ et Medicinæ Doctoris, Christianissimi Regis Caroli IX., Serenissimæ Reginæ et Invictissimi Ducis Brabantiæ, Andium etc., Comitis Flandriæ etc. Consiliarii et Medici, Opera Omnia Medica et Chirurgica. Hac postrema editione à mendis repurgata, methodicè disposita, paragraphis distincta, notis marginalibus, et authorum testimoniis aucta, hinc inde annotationibus illustrata, prodeunt e musæo* JOANNIS VAN HORNE, M.D., Anat. et Chir. Prof. Ordinarii in Acad. Lugd. Batav. Lugduni Batavorum ex officina Danielis et Abrahami à Gaasbeeck, 1660. 12mo.

IT is a remarkable circumstance in the medical history of the present time that a remedy, which only a few years since was familiarly employed by nearly every American practitioner, has so far gone out of use that very few and exceptional cases are now met with in which it is resorted to by the physicians or readily accepted by the people. Thirty or forty years ago the case was different; as both the people and the medical men were alike prone to avail themselves of phlebotomy as a cure or as a safety in even trivial disorders or accidents. A physician who was opposed to blood-letting forty years ago, was looked upon with distrust—as a timorous and unreliable practitioner; whereas he who now resorts to his lancet boldly, is regarded as a Sangrado, and, by some persons, looked on as given to vampirism; for to bleed a sick man has been charged as an act of vampirism.

Seeing, then, that so great a revolution in private and professional opinion has taken place, it would appear to be not ill timed for us to inquire into the facts, and endeavor to learn whether we moderns are wholly in the right, and our predecessors wholly in the wrong; we, in almost wholly ignoring—or, to say the least, neglecting to use a remedy of acknowledged efficacy, and they, in applying it to a perhaps dangerous excess.

It is certain that the use of blood-letting, as a means of curing or of modifying the course of diseases, is one of those of the extremest old date. Hippocrates, the father of medicine, made a frequent use of it four hundred years before the Christian era; and as he is justly to be considered as the general representative of medicine for the entire period from the war of Troy, in A.C. 1100, down to his own date, it is certain that we have, in phlebotomy, one of the earliest resources of civilization against the pains and the risks of disordered health. Certainly mankind have had recourse to it under this apparent necessity, for at least three thousand years; and it is curious to inquire why they have of late so generally discarded it.

The venerable Dr. Jackson, of Boston, so late as March of the present year, has published a volume containing "Another Letter to a Young Physician," etc., in which, at page 55, speaking of the change as to venesection, he says:—

"I can hardly say just when it began. For the last *twenty years*, at least, venesection has almost gone out of use among us in medical, if not in surgical practice." * * * "Nevertheless I feel persuaded, from my own observation and experience, that the use of the lancet should not be altogether abandoned," etc.

Our own experience in medicine now extends over more than fifty-two years; and we coincide with Dr. Jackson's opinion that the change occurred about twenty years ago, or about the year 1840; and that the change took place without any known excitement, and silently and gradually; without any notorious discussion, or any conflict of opinion concerning it among leading men in the profession.

Having grown up and having participated in the transactions and in the times when the use of venesection was carried to the greatest extent in the United States, and having lived to witness its almost entire abandonment by both people and physicians, we often ask why this change took place, and whether similar changes in regard to it are recorded in the history of medicine; and if so, then whether it may reasonably be expected that the remedy shall again come into favor, and afterward decline as the cycles of opinion shall roll around.

It was stated above that the father of medicine, who, as a representative man, may be regarded as a bright reflection of the lights of the ages that preceded his own, does show us in his works that for more than a thousand years before our era, phlebotomy was in use as a means of cure. Yet, within one hundred and fifty years after his death, a violent opposition to it was set on foot by Chrysippus, and a war to the death waged against it by his pupil Erasistratus, at the Alexandria school, and by his disciples and followers.

It was against this heresy in medical practice that Galen raised his voice; and it is to Galen that is due the credit, if any, of re-establishing the lancet in the public favor. The eloquence and learning of Galen placed him at the head of the whole medical body in the world, and the reputation and authority he attained held on in one steady, uniform course of dictatorship during some fifteen centuries. This great authority of the physician of Pergamus did therefore uphold our remedy in the confidence of the medical people and the populations everywhere.

It was more than three centuries subsequent to Galen that Oribasius published his works on medicine, and those works show that the Emperor Julian's archiater was as bold a bleeder as Galen himself, or as our illustrious American, Benjamin Rush. In the long course of this Galenical supremacy, there arose many men of independent judgment, who, though acting under the dominion of his authority, might make use, some more, some less, of phlebotomy in their practice; but the employment of the lancet, so far as I can learn, was never so generally abandoned as it began to be about the year 1840; and it is equally true that as a remedy, it was never carried to such excess as it was carried to from about 1580 to 1750, when it fell off considerably, to be reinstalled in favor, particularly in the United States, by the lectures and publications of Dr. Rush.

The writer of this had the happiness to be an attendant at the course of lectures in the University of Pennsylvania of Drs. Rush, Physick, Chapman, and Dewees, all of whom were bleeders of the highest mark. Perhaps no man of modern times was a more profuse bleeder than Professor Physick, who, in one of his lectures on concussion of the brain, related this anecdote of his master, John Hunter: a man who had fallen from a height was brought into Mr. Hunter's ward insensible from concussion, and when Mr. Physick reported the case to the distinguished surgeon, he asked: "Shall I bleed him, Mr. Hunter?" "Bleed him, sir?—bleed him in concussion of the brain, sir! No, sir: cover his head with cold wet cloths until he reacts, and then bleed him, sir; bleed him to death!"

In Dr. Physick's time no person had become acquainted with the antiphlogistic properties of nitrate of silver as a remedy for the inflammations of the conjunctiva. It was then held as a reproach to any practitioner who should allow an eye to be lost from chemosis, or any form of acute ophthalmia. At page 134 of Dr. Jackson's letter, above cited, in relating the medical case of Prescott the historian, who in his early youth was attacked with a rheumatic chemosis, that practitioner tells us that, in the course of five days, the youth lost, "by general and local bleeding, more than seven pounds of blood;" a fact that serves to show how prone our people were to rely on the curative powers of the lancet, even at Boston, where the practice is now almost ignored.

As an exemplification of the habits of practice in Philadelphia, I shall relate that about forty years ago I attended a stout woman here, who was near fifty years of age. She was attacked with an alarming inflammation of the conjunctiva, which assumed the form of chemosis, and threatened to destroy vision by producing opacity of the cornea. Having been instructed by Prof. Physick, and having adopted his views of treatment for such ophthalmia, I bled the patient very freely, and made use of other measures as juvantia. The bleedings were repeated day by day, but without making the least impression on the diseased eye. Still, tortured by the thought that I should inevitably lose the eye, I persevered in applying the *remedy;* so that at length she fainted so badly as to cast me almost into despair. Under these circumstances I visited the Professor, who kindly engaged to meet me in consultation. I had laid before him the whole history of my proceedings, and told him that I had carried out his instructions to *outrance*, and dared not to repeat the operation. Dr. Physick paid the visit. Light was momentarily admitted to the darkened chamber. He touched the wrist, depressed the lower lid, and said, "that will do." When we were met in another chamber, I said: "You see, sir, that I have bled the woman as much as it was possible to do without danger, and now I shall lose her eye." "Not at all, not at all, sir," he said; "you should bleed her again, and you should order the bleeder to return to-morrow morning to repeat the operation, and when that is done tell him to come back again the next day if the eye should not be recovered." Poor Mrs. Smith was duly bled again, and Mr. Ripperger came and bled her on the following morning; but on the third morning, as the ophthalmia was clean gone, she lost no more of her vital fluid. Nor did she afterward suffer the least inconvenience from such a violent method of cure. Indeed, I have sometimes been almost convinced that a woman cannot be injured by blood-letting, seeing that any one would stop, in time to avoid dangerous consequences, however bold and uncompromising he might be in the use or the abuse of this great remedy.

Now that blood-letting is so much gone out of fashion in the world, both European and American, the question ever comes up, why is it that in reasoning by induction, which consists in collating and comparing and judging from the facts, we should in our modern induction cast aside all the facts accumulated during more than three thousand years of observation and records, and rely solely upon those observed in the short space of the last twenty or thirty years only?

Some persons have argued that the discharge of venesection from the common routine of medical practice is attributable to the example of the Hahnemannists; but I consider that conclusion to be too humiliating to our medical philosophy to be deserving of our regard. It is far more probable that the silent change in professional opinion is due to the discovery of the cell-life by the histologists, and that the new doctrines of absorption by cells, and the consequent new lights we enjoy on the subject of nutrition, are the real cause of the lapse of public opinion on this topic. It may be, however, that the revolution is an inscrutable consequence of what Sydenham denominated a change in the constitution of the air, one in which diseases of an inflammatory type do now rarely call for the interposition of phlebotomy as a remedy, which, in disorders of the same name, would be incurable without the loss of blood. If this be the true interpretation of the curious incident in our medical history, the physician is now living who shall see the lancet reinstated in all its authority and usefulness; and this is what I now believe. I say I believe this, because I cannot understand that human opinions upon so economical a matter as a thirty-century-old habit and practice, can be changed out of mere caprice, and much less that our boasted modern wisdom so infinitely excels that of our forefathers as to warrant us, reasoning from induction, to fly in the face of Bacon, and trample under foot more than 10,000 per cent. of the facts on which that induction ought in fairness to be founded.

LEONARD BOTALLI lived in an age when, from some cause now unknown, the doctrines of Hippocrates, of Galen—nay, of the long-passed centuries—ceased in a measure to be respected, and hundreds of sick and wounded people were permitted to languish in pain and to die, because society, and the doctors as well, had begun to deem it unfashionable and unwise and rash to take blood as a means of cure.

Botalli was born at Asti in Sardinia, some thirty or forty miles southeast of Turin. It is not known in what year he was born, but his tract, "De Curatione per Sanguinis Missionem," was first published at Leyden in A.D. 1580; and in the copy before us, whose preface is dated by him at Antwerp, in the month of June, 1582, he says, at page 336, that he "has now been in the habit of bleeding, as a means of cure, at least forty years;" from which we may conclude that he was in practice as early at least as the year 1542. It is probable that he might have been as much as twenty-five years of age at the commencement of his medical career, which will carry the date of his birth to near 1520, about the time Hernando Cortez was preparing his expedition for the conquest of Mexico.

We have no record of the date of his death, but he was probably about sixty years old when he gave his interesting book to the world. Botalli had enjoyed great advantages in the means of his early education, and that he made a good use of them will appear to every reader who will observe the lucid order of his thoughts, the purity of his style, the various and extensive learning which everywhere distinguishes his productions, and witness the strict integrity, truthfulness, and honor that mark his whole course. No man in our ranks was more charitable to the poor, nor more open and undisguised in the expression of his convictions. In short, Leonard Botalli is a name that adds grace to our annals, and, though dead so long ago, he yet lives and speaks to us in a volume that is become a holy classic in medicine.

The entire tract is comprised in forty-two chapters, that occur in the following order, with their proper headings:—

Chap. I.—How the ancients employed blood-letting. The diseases in which they used it, and those in which they declined to use it. The nature of the blood.

How it is formed. In what way it is useful, and in what way useless in the body.

Chap. II.—Blood-letting, when required, is useful in the diseases of both children and aged people.

Chap. III.—Is blood-letting suitable in cases of pregnancy?

Chap. IV.—Venesection is adapted to fluxes from the vessels.

Chap. V.—Blood may be drawn in the commencement of exanthemata, in parotis, or other critical evacuations.

Chap. VI.—How blood should be taken in the treatment of actual menstruation, or during the lochia, in epistaxis, or other critical hemorrhages.

Chap. VII.—Is venesection useful in plague?

Chap. VIII.—Blood-letting necessary in cacochymia.

Chap. IX.—Whether bleeding is adapted to the treatment of flatulent pain.

Chap. X.—Bleeding necessary in the case of convulsions.

Chapter XI.—In epilepsy, suffocation, and other disorders of the kind, blood is to be drawn.

Chap. XII.—In catarrhs and obstinate pains, bleeding to be had recourse to.

Chap. XIII.—Phlebotomy to be used in dry cough.

Chap. XIV.—Venesection useful in pains in the abdomen.

Chap. XV.—Whether phlebotomy is suitable in dropsical persons and in inveterate quartans.

Chap. XVI.—Whether bleeding is adapted to recent quartans.

Chap. XVII.—Whether in fevers tending to tabes and emaciation blood-letting is an appropriate measure.

Chap. XVIII.—*Post superfluam venerem*, phlebotomy not to be avoided if the indication for its use exists.

Chap. XIX.—None but the ignorant hold blood-letting to be injurious to the sight, or that a first bleeding has greater powers of curing than a second, or a third, or a hundredth. The indications in venesection are—

Chap. XX.—Indications derived from the nature of the disorders;

Chap. XXI.—Indications from the part affected;

Chap. XXII.—Indications from the habits and movement of the disorder;

Chap. XXIII.—Indications from the season of the year.

Chap. XXIV.—Of the quantity of blood to be let in proportion to the strength.

Chap. XXV.—Of bleeding ad deliquium animi.

Chap. XXVI.—Explanation of the indications employed by Hippocrates and Galen in blood-letting.

Chap. XXVII.—Indications derived from the tint of the blood and its consistence.

Chap. XXVIII.—The opinion of the author as to the quantity of blood in the body, and how much may be taken, or spontaneously effused, without loss of life.

Chap. XXIX.—He shows us by numerous examples, as well out of Galen as from his own experience, how much blood may be drawn.

Chap. XXX.—By stating cases and their cure, he endeavors to show how much blood may be drawn.

Chap. XXXI.—Whether small and few blood-lettings are proper in long and obstinate disorders.

Chap. XXXII.—Objections of his adversaries refuted and explained.

Chap. XXXIII.—Shows by many instances how much blood is formed daily in the body.

Chap. XXXIV.—Repels the peurile notion of those who attribute greater usefulness to a first bleeding than to subsequent ones.

Chap. XXXV.—Whether the first blood that is formed is better than that subsequently produced.

Chap. XXXVI.—Physicians, both ancient and modern, have erred rather in deficient than in excessive use of bleeding.

Chap. XXXVII.—Whether bleeding should be performed before or after purging remedies have been employed. Whether phlebotomy may be used without regard to purging either before or after it.

Chap. XXXVIII.—What is to be attended to while bleeding the patient, and what is to be done in case of fainting?

Chap. XXXIX.—In performing the operation, what mode of opening the vessel is most in use?

Chap. XL.—What vein is preferably to be opened, in various diseases?

Chap. XLI.—Of arteriotomy.

Chap. XLII.—Of local bleeding.

Now it rests with the reader to cast his eyes over this series of headings to Botalli's chapters, which may serve at least to show, like the carte at a table d'hote, what is the bill of fare in his reach; but, as a physician retired from the busy scenes of practice and retaining only an affectionate interest in a profession to which more than a half a century was assiduously devoted, the writer could not repress a wish that every student of physic in America might be held to study Botalli's tract as a text-book, rather than the cold and formal tract of Celsus, which has been repeatedly preferred as a horn book for medical students.

It is the fashion of the day to put aside the old and standard authors in medicine in favor of the modern monographs, the bodies of physic, and that gallimatias of journalistic literature so pregnant of hope and promise while so barren as a substantial and refreshing fruit. Yet it is difficult to conceive that an honest and intelligent observer like our author should be sent to his grave to be forever after sneered at as a man whose words, whose efforts in behalf of the true and the good, deserve nothing but the contempt of the living. If a true and faithful heart did beat in man's breast, animating and developing a great and useful intelligence assiduously employed in the interests of mankind, there seems to me to be a cold and a base ingratitude in them who treat their memory with indifference or scorn; and I cannot suppress the feeling of disgust that arises when I hear a professional jackdaw glorifying himself because decked in the feathers of half-a-dozen text-books, he plumes himself upon the glorious nineteenth century—the age of telegrams and steam—but scarcely therefore the age of philosophy and common sense.

Botalli studied medicine both at Pavia and Padua, and had for his masters the most illustrious teachers of the time, as Cardan, Frigimelica, Lanfranc, Falloppio, Trincavelli, and other learned philosophers, who had stored him with that wisdom and knowledge which served as the seeds to a larger growth which sprung up in the service and at the courts of Louis IX., and of the Prince of Brabant and Flanders and Duke of Anjou. He appears to have passed his whole life amid the most brilliant society of the saloon and the camp, and to have thus attained to that elevation in his intellectual and moral sense that belongs to the very highest training. If such a man relates with candor and truthfulness the story of his experience as a physician, it is as worthy of acceptance and credence to-day as if it had to-day been uttered by some celebrity of London or Vienna, of Paris or Berlin. But our intention not being to set forth a special plea for Leonardo, let us turn to his book and allow him to speak to us from his own mouth, by citation of his own words, *de curatione per sanguinis missionem.*

The second chapter is devoted to the inquiry whether blood-letting is admissible as a remedy in the diseases of children and of aged people, a practice condemned by Galen, yet deemed by our author not only safe but useful in the incipient stage of violent disorder and where the patient is plethoric. In such case, an aged patient may suffer as much as one pound of blood to be drawn from the arm, to be repeated in the course of six hours by another bleeding to the amount of half a pound. In case such second bleeding should be determined on, it ought not to be done without having given some proper nourishment.

Botalli asserts that for an aged man not in good health, and affected with impurity of the blood, it is neither superfluous nor hazardous to lose blood by venesection from four to six times in the course of one year, and he has no hesitation in saying that the new productions of the vital fluid that take place most readily after hemorrhages constitute a real advantage to a man in years.

As to this notion of Botalli concerning newness of blood, it should be observed that the daily ration of a man is what is regularly required to make up for the daily waste of the old blood in acts of nutrition and of secretion, and that a man who has not been bled requires his three-quarter pounds of pork, or his pound of beef, and his quota of bread and beans, as regularly as if he had passed through the hands of the illustrious Sangrado himself. Nevertheless, there lurks, perhaps, in every physician's mind a latent idea, that the fresh newly-developed and diffluent blood that fills the vessels of one who has been exhausted by great losses from wounds or from fever is in fact more active and more useful; as if it had been drawn from some *fontaine de jouvence.*

In the third chapter, on the question whether bleeding is a suitable remedy in the cases of pregnant women, he quotes Celsus as an advocate of the practice, while he shows both Hippocrates and Galen to be in the opposition. So great was the authority of these two names, that the medical people of his day felt impelled by it to refuse blood-letting in pregnant women, even when laboring under the gravest and most acute disorders. It is in this connection, and to show how needless it is to be bound by a dogma, even when issued from the school of the father of medicine, that he relates the history of Lady Drury's case, which came under his care while in London, as one of the suite of the Duke of Anjou. This Prince of Brabant and Flanders and Duke of Anjou had gone to London with a view to solicit the hand of Queen Elizabeth, so that Leonardo was honored by being made acquainted with the sovereign, and by conversing with her relative to the illness of one of her favorite ladies of the court, the Lady Drury.

This occurred, he says, in "the course of the present month of June, at London." The Lady Drury was in the seventh month of her pregnancy, and was at the time suffering from an obstinate tertian fever, attended with most excruciating pains in the hypogastrium and the right hypochondrium, and with a nasty jaundice and a pressure downward of the fœtus toward the pelvis.

In the presence of the French Royal Duke, Queen Elizabeth, who was much attached to the Lady Drury, asked Botalli to visit the sick lady, saying she thought it possible that bleeding might be a proper remedy for the disorder, and requested him, if he should be of the same opinion, to persuade her at once to submit to the operation.

Botalli found her complaining grievously, and in addition to the symptom already mentioned, having very dense and inflamed urine, and immediate expectation of premature confinement. The patient was twenty-eight years old, and stated that she had been seized two months before with a simple tertian, which soon denegerated into a double tertian, and then became a continued fever, such as he actually saw it to be. The pain and sleeplessness were very great. She said that purgative medicines had been prescribed to her from which she felt no better, but worse; that the learned Dr. Bailey, physician to her majesty, (Qu. Walter Bailey?) had, at the commencement of the illness, spoken of bleeding her, but as he was in consultation at the time with another physician who dissented, it was not done, though the practitioner had agreed to yield to Bailey's superior authority. This disagreement of her medical attendants had alarmed her so that she would not agree to the operation at the time, after which the proposal was never renewed; that the doctors had at first thought of bleeding her, and Dr. Bailey, her majesty's physician in ordinary, was in favor of doing so, but seeing that the consultation were not unanimous on the subject, and inasmuch as it was commonly conceived to be dangerous to let blood from pregnant women, particularly at the seventh month, the operation had not been performed, because she was frightened about it on these accounts, and because Bailey having gone with the queen to Richmond, nothing more had been said about it in his absence. Botalli told her the only proper remedy now was venesection, both in her own interest and in the interest of her child; that if abortion should take place it would be not because she was bled, but because she was ill. To this opinion Lady Drury assented, saying that her mother, Lady Stafford, who was always in attendance on her majesty, had, by the queen's command, repeatedly desired that she should have it done. Botalli said, "In such a disease as yours, so serious, so obstinate, with such a habit of body, and so vitiated a state of the blood, it is most evident that blood-letting is demanded, but not excessive, nor for once only, but should be repeated several times in the course of several days; still, as Dr. Bailey and the other physician are now absent, nothing can be immediately done, especially as there is plenty of time yet for consultation." The consultation met in the evening, and after a conference, in which Bailey regretted that it had not been done earlier, he now feared it was too late to do any good, though he had no notion it could be injurious to the lady, though in case of the threatened abortion taking place, the doctors must inevitably be discredited, seeing the general opinion of society on this subject. The end of the meeting was an agreement, so that, the next day at nine in the morning, after a very bad night in which she did not sleep a wink until near morning, they drew away ten ounces from the basilic vein, scarcely one-eighth part of which appears to be blood, and that *perust*, covered with a thick coat of size, and all the rest serum. The pains were much lessened by the operation. At 2 P.M. the vein was again opened, and about six ounces taken which resembled the former portion. The fever was diminished, the bearing down was less, and everything looked favorable. Botalli advised that she should be bled again in two days, which, however, was not done because she seemed to be easier, to his regret; yet, when in the course of eight or ten days afterward the bleeding was reiterated, she felt so much relieved that she told him, smiling, how a physician, whose name she would not give, had called upon her, half an hour before Botalli's first visit, to say that, hearing Botalli was to see her with a design of letting her blood, etc., "*pietate ductus*," he had come to let her know how dangerous a thing it was, and how completely opposed to the best authority and practice, to take blood from pregnant women, and to warn her of the danger of being bled once, much more twice. So my Lady Drury dismissed him, with a refusal to allow him to be present at the consultation, though he was considered one of the most learned physicians in London. She told Botalli that she had kept this incident to herself, lest if she had informed him of it, he might, for fear of calumny, act with less independent judgment. Botalli here breaks out on the impudence of this doctor, and proves the steady good sense of his patient who had thus been preserved, and who assured him that hereafter she intended to resort to bleeding when sick, whether pregnant or not, and in spite of the maxims of the doctors.

The rest of this interesting chapter is taken up with accounts of and references to numerous cases in which he had used the lancet for women in gestation. He tells how that at Blois, being invited to supper by the Duchess of Nemours, that lady remarked that he (Botalli) had inspired the minds of ladies *enceinte* with courage, and had given to the physicians also no little confidence in the use of venesection; but a learned doctor who was at table observing that there was nothing new in the practice, the Duchess answered, "You may say what you will about your ancients, but the fact is that the general use of bleeding as a remedy in France these ten years past, and which is so much vituperated by many doctors, is due to Botalli;" and the Duke of Guise added that he had repeatedly said the same thing to the doctors, and Botalli insists that for fifteen years past he has promoted the employment of this great resource both by example and precept, both verbal and written.

It is not expedient here to follow the author in his argument in favor of the use of the lancet in the management of diseases of pregnant women, because, in the first place, such an exposition would be tiresome to the reader, and in the second place, because we presume there be but few American physicians of some twenty or thirty years' experience in the practice, who remain ignorant of the former custom in this country to bleed nearly every pregnant woman at least once by way of precaution, and as the safest and readiest of all prophylactics for the gravid. The same persons, probably, are as well aware as the present writer, that great inconveniences have followed the abandonment of so salutary a custom, since they cannot ignore the fact that most elaborate investigations have of late years been called for into the nature and causes of those renal and phlebitic affections that are now so rife and dangerous, and we add so fatal, because the new practice of delaying the use of venesection has invaded even this country, to the overthrow of those health-giving and life-preserving principles which, though they sprung not originally out of the fruitful and active brain of Leonard Botalli, yet were so plainly and so eloquently set forth by him that they were for two centuries enabled to triumph everywhere over the insane objections of the timid followers of Chrysippus and Erasistratus.

Relieved as we are from the practice of an art to which fifty-two laborious years had been sedulously devoted, and withdrawn from the scenes of active operation, we feel that we ought to be able dispassionately to review the long and wearisome track on which we so perseveringly toiled. Well, such a review compels us to declare that the practice of physic is by no means so efficient, so beneficent and conservative as it was in this country thirty or forty years ago; and we are firmly persuaded that the same thing is true of Europe—where medical men seem to have tumbled into the deep slough of the chemicalists, because they will believe the living body to be some sort of an alembic, in which life, function, even thought, is to be distilled, and the result to be modified by a *quantum suff.* of salt, sulphur, and mercury, phosphorus, arsenic, *et id genus omne.*

We may refer the reader to the close of this chapter for an account of the way in which Botalli's brother treated his two daughters, both of whom were *enceinte,* and suffering with acute fever and pains of the head and back. This gentleman, who seems to have been a hearty convert to the lancet doctrine, took away from each lady three pounds the first day, and as much on the second—abstracting on each occasion two pounds in the forenoon and one in the afternoon. By this method the ladies were completely cured on the fourth day, without the least damage to either mother or child.

Chap. iii. is devoted to the discussion of the influence of venesection in diarrhœa and dysentery. To show how favorably it acts in these disorders, he cites several cases at length in which he happily succeeded in curing people far gone in disease. He knew that dysentery was inflammation of the gut, and founded his practice on that idea.

Eruptive disorders and cystical tumors and evacuations are discussed in chapter v. with the same result of proving that abstraction of blood is one of the most useful and safe of methods; and in the sixth chapter he enters upon the question whether bleeding is allowable during the catamenia, in epistaxis, or in critical hemorrhoidal hemorrhages; all of which questions he examines with much discreet wisdom, and shows that the existence of any one of them is no bar to the indication, where that indication can be made out.

Botalli devotes his seventh chapter to the discussion of the question as to the treatment of plague by blood-letting; and he concludes that there is no form of that frightful disorder in which his great remedy is inapplicable, and that it is even superior to all others.

In the third section of this chapter he puts forth a statement of the opportunities he had enjoyed of learning what was the general opinion on this great subject. "If you ask," says he, "how I came to know all this, I answer that I know very well that the medical men from Galen downward, and all the physicians of my own day and acquaintance, whether my own teachers, or men of Pavia, Milan, Padua, and Venice, in France, England, Hanover, and Flanders, were all very sparing in blood-letting—rarely taking more than three vascula (nine ounces, or at most ten ounces) at one time;" and he shows that the bolder practice had been introduced and made known by his writings and by his brother's. This chapter on plague contains the history of Capluan's case, in which that individual was taken to Botalli's house and cured of a dreadful attack of plague, with vibices and buboes and abscesses, by the most audacious venesections. The case, however interesting, and however well calculated to set forth the safeness and advantage of bleeding in the case of plague, is too long for insertion in this place. Doubtless many persons, in perusing the curious relation of Capluan's case, would at once pronounce the doctor a madman and the patient a fool; yet it must be admitted that, if any medical writers can be relied on for veracity and science without exaggeration, Botalli is one of that class; and also that facts are stubborn things, that will not lie any more than mathematics will lie—which they cannot do, though mathematicians and doctors can.

Capluan lost by venesection, in his attack of plague, 136 ounces of blood at eight bleedings; and to that bold practice he, in a grateful letter to Botalli, attributes his escape from death. A second letter, of later date, states he is in perfect health; in it he observed, Botalli had no right to say that the doctors from Galen downward were timorous bleeders, for he had only to read the eighth chapter of Book I., Synopses of Oribasius, to see that that colleague was not afraid to bleed twice, or even thrice, in the same case, nor to carry the subtraction to the point of a deliquium animi. To read instances, cap. ii. and iii., *Collectiones*, lib. vii., will show him a sufficiently prompt bleeder, though an obedient servant of Galen as to the injunction against venesection in children under fourteen and in old people. In the third chapter, *de partita sang. missione*, he directs us to divide our bleedings so as to take the proper quantity for the cure of the disease in divided doses for patients who, though weak, may be esteemed to demand venesection as a remedy; while in the strong, and in early stages and acute disorders, he was a resolute bleeder. But we are not writing a retrospective review of Oribasius, who was physician in ordinary to Julian the apostate, and flourished in the closing years of the fourth century.

Chapter viii. treats of cacochymia and its cure by the remedy. Chap. ix., Is venesection a suitable remedy in flatulent colic? Chap. x., Convulsions to be treated by blood-letting. Chap. xi., In epilepsy, and in strangulation, etc. Chap. xii., In catarrhs and obstinate pains. Chap. xiii., In dry cough. Chap. xiv., In pains of the belly. Chap. xv., In dropsies and inveterate quartans; and on this point he speaks feelingly, because he had recovered his own health ten years before, when in great danger from a dropsy that was brought on by a quartan ague. His good fortune was due to his having been, at short intervals, three times bled, to the amount of from one pound to ten ounces; and this step he took at the earnest solicitation of his brother, who seems to have been as characteristic a Sangrado as Botalli himself.

Chap. xvi. treats of the applicability of the remedy in recent quinsy, and gives cases to show the good results. Going now forward a chapter, we find him discussing the question whether blood-letting is injurious to the sight. In support of his views, he cites the case of the Cardinal de Biragua, who was keeper of the seals for Charles IX. in 1570 at Paris. He was one of the formers of the plot for the St. Barthelemy, cardinal in 1578, at the instance of Henry III. † 1583. This man's health and sight were greatly strengthened by the repeated bleedings prescribed by Botalli. But our author, having a serious affection of the sight, bled himself enormously, and perfectly recovered from the disorder.

The twenty-fourth chapter treats of the quantity of blood to be drawn, *pro ratione virium*, and the twenty-fifth of bleeding *ad deliquium*. The twenty-seventh chapter shows that a good indication as to the quantity to be drawn may be taken in the tint of the stream as it flows away; but he is very careful to insist that the color of the flowing stream is not solely to be depended on, and we ought to judge of the case by inspection after the basin has rested awhile; and the judgment to be formed of the quality of the blood will depend upon the form of the vessel into which it is received—if in a shallow and wide dish, it will cool and coagulate so rapidly that we shall not be able to discover the various parts of its constitution—everything being compressed and compacted. But if the stream is received in a deep and narrow cup, it will coagulate more slowly, and thus give time for the more complete separation and disintegration of its several constituents.

Chap. xxviii. treats of the quantity of blood that is contained in an adult human being, and at page 272 he cites the opinion of Avicenna, that the sum total amounts to twenty-five pounds, of which experience shows that seventeen pounds may be lost in the course of a single day, from wounds or other causes, without necessarily causing the death of the patient. [This day is April sixteenth, and yesterday I saw Mrs. ——— at Philadelphia, who had just recovered from her menstrua. She told me that she had used six napkins a day for seven days, large ones, which yet were not sufficient to prevent an overflow on to her feet. Now here are

40 or 42 napkins, each of which would hold not less than 6 drachms = 240 = 30 ounces. Very well; but this lady would make water at least 5 times a day, and never without a considerable quantity of the fluid that is always being collected within the vagina, restrained by the sphincter vaginæ, and which must be assumed to = 35 times 1 ounce, or 35, which, + 30 ounces, ut supra, = 65 ounces lost in this menorrhagia, and which, to all appearance, had not in the least affected her pulse, her color, or her strength; and this is a case that I have witnessed a hundred times; so that I do positively know that a woman may lose fifty or sixty ounces of blood in a menorrhagia without sensibly interfering with her health and security.] Leonardo goes on to express some surprise at Avicenna's statement, because, as he thinks, there are few people who could bear the loss of ten pounds at one draught and live, though he has seen some persons lose ten pounds in the course of a single day without loss of life, and many he has known to lose twenty pounds in the course of one week. It is easier, says he, to lose thirty pounds in the course of eight days, provided the strength is meanwhile supported by proper food, than for the strongest man to lose ten pounds in a single day without in the mean time taking any nourishment to keep up his strength.

Our author devotes the twenty-ninth chapter to an inquiry concerning the quantity of blood that may be taken away by venesection, with examples out of Galen's experience and his own; and that he was not at all attacked with what he calls hæmaphobia, one can see by reading the closing section of this chapter. This subject is continued in his thirtieth chapter, wherein he relates the practice of his brother, Francis Botalli, a surgeon, who appears to be quite as much *Sangrado* as Leonardo himself. It is curious to see how audaciously Francis bled his young daughter, whom he spared no more than he spared other folks; for it was his way, *sæpissime libras quatuor et quinque cum maxima ægrotantium utilitate detraxisse*, p. 284.

Francis had a daughter seventeen years old, who, being seized in the dog-days with an ardent fever, at a time when the plague was prevailing in the country, was bled on the third day of the disease to the amount of thirty-two ounces. He took another pound in the afternoon. On the following day, seeing that the disease was aggravated in violence and accompanied with intense headache, he drew off another pound; and on the succeeding day, the disease still holding its violent course, so that he almost despaired of her life, he anxiously doubted whether he might venture to bleed her again. At length he acquired confidence to that degree that he bled her to the amount of one pound, which gave her a good night and freed her from the disorder. It happened, says Francis, that two hours after this last operation, she fainted so badly as to greatly alarm him; but when he had given her some good meat broth and yelk of eggs, the weakness disappeared and she was perfectly restored.

In the thirtieth chapter the author says: "We have shown, with sufficient clearness, that most physicians have erred in letting blood both too late in the disease, and in quantity too small, and we shall now proceed to inquire whether they have, on the contrary, erred by any excesses." He considers that in general they bleed too sparingly, though some of the followers of Erasistratus and Thessalus, after being converted from their sectarian views, were accused of having destroyed people by the extravagant use of the lancet. Hippocrates was not the inventor but only a follower in a practice that long preceded his epoch, and was, so to speak, a timid bleeder. It was to the influence of Chrysippus and Erasistratus that was due the almost entire cessation of the use of venesection in medical practice, until Galen, by his example and writing, reinstalled it as a method of curing diseases.

Botalli makes a considerable mistake in stating that whereas the practitioners of physic who adopted the fears and objections of Erasistratus were prevented by their deference to his great authority, down as low as the era of Galen, and though that illustrious physician not only defended the views of Hippocrates, but absolutely resuscitated and restored to life and activity the use of venesection, that had, for so many years, been completely dead and buried by Chrysippus and Erasistratus, yet even after him the habit of obedience and a still lingering hæmaphobia prevented the successors and even the admirers of the Pergamenian from bleeding freely and boldly in their practice. We say he made a mistake here, because it is certain that Oribasius, who was archiater to Julian the apostate, and who was induced, by the request of that emperor, to draw up and publish his great works on medicine, was a bleeder of the boldest sort, as the reader may learn in both his books of Synopses and in his Collections, in the 4th chap. and 7th book of which he recommends venesection to the amount of ten ounces, to be followed soon, if requisite, by another abstraction to half that amount, which agrees well with the ideas of most moderate men of the present day. He says that many physicians refrain from venesection to a proper extent, out of fear, not of the evil consequences that might ensue to the patient, but to themselves, on account of the public sentiment, which was mainly in opposition to liberal detraction of blood in the sick; but he assuredly believes that 100,000 persons are permitted to die of diseases from want of early and sufficient blood-letting, to a single individual who might perish from excess of it.

It would be calling too freely upon the reader were we here to lay before him a considerable series of the very interesting and well drawn up cases with which the book is replete. Perhaps it will be deemed that we have already dwelt too long upon the notions of a doctor who died not less, probably, than two hundred and seventy years ago. But to peruse the pages of so excellent a physician and so worthy a gentleman is a thing impossible for us, without being convinced that he is one of our best teachers and guides in practice. Very lately we read again, perhaps for the twentieth time, the cruel satire on blood-letting by Le Sage, contained in his account of Gil Blas's connection with Dr. Sangrado and the licentiate Sedillo of Valladolid.

Yet a satire may be more bitter than gall or wormwood, though the subject of it be pure as light and sweeter than the honey-comb, and while it is impossible to suppress a smile at the picture of Sangrado's practice, we are not a whit the less inclined to smile when we think of those who at the present day are as much hæmophobic as the sectarian Chrysippus, and who reason on blood-letting as if the daily incidents and events of a medical man's life did not bring often before his eyes the evidences and the proofs that men and women may suffer enormous losses of blood, and by a long and healthful subsequent life show that such hemorrhages, as the artificial or spontaneous, are safer as remedial agents than the potent drugs and medicines that with the utmost complacency are prescribed and taken, with a view to bring about some mysterious chemical result under which the crasis not of the blood only, but the whole living solids, is to be restored to a true physiological normality.

Our own opinion on the subject coincides with that of good old Dr. Daniel Le Clerc, author of the History of Medicine; and here is where he says, that—

> "Having gone over the ground covered by what is properly called the first age of medicine, it would seem that but little can be gleaned out of all that old time of really practical and useful medicine: all is fabulous and uncertain, or at least extremely confused; nevertheless, if medicine consists rather in its effects than in its doctrines, and if the discovery of remedies is more important than any reasonings whatsoever concerning diseases, we shall be obliged to admit that those old time peeple were acquainted with nearly everything deemed really essential to be known by modern physicians throughout Europe; that they employed nearly all the fundamental remedies, such as are most relied on at the present day. All physicians, with a very few exceptions, look upon bleeding and purging as the most universal of remedies."

We are not of the class of those who abhor venesection, and who reason upon artificial loss of blood as a real attack upon the vital principle, as was the idea of some of the ancients. The practice of physic is not improved by this change. Many a valuable life is placed in peril by the new fashion of oh-oh-ing and pooh-pooh ing the shocking hint that a vein might perhaps be with advantage breathed.

As a dispassionate, and now disinterested spectator of the scenes of medical practice, we look with regret upon the present disfavor of a remedy that has received the sanction of mankind for innumerable ages, and which, though under some mysterious and unknown influence, now nearly banished from the world, must inevitably again rise into favor among men, and again serve as one of the strongest of the few feeble barriers we are able to raise against the assaults of pain and the threatening approaches of death.

